# Artificial intelligence for the next generation of precision oncology

**DOI:** 10.1038/s41698-021-00216-w

**Published:** 2021-08-18

**Authors:** Pedro J. Ballester, Javier Carmona

**Affiliations:** 1 Cancer Research Center of Marseille, INSERM U1068, Marseille, France; 2 Institut Paoli-Calmettes, Marseille, France; 3 Aix-Marseille Université, Marseille, France; 4 CNRS UMR7258, Marseille, France; 5 Nature Medicine, New York, NY USA

**Keywords:** Predictive markers, Predictive markers

The concept of precision oncology involves the prescription of therapies that target the molecular driver alterations of an individual patient’s tumor. This treatment paradigm has been accelerated in recent years through increasing integration of molecular profiling approaches into mainstream clinical oncology, and the approval of a variety of molecularly targeted agents that have improved the clinical outcomes of patients across multiple cancer types. Targeted therapies such as trastuzumab or vemurafenib have become the standards of care for patients with HER2-expressing breast cancers or BRAF-mutant melanomas, respectively, and immune checkpoint inhibitors recently received tissue-agnostic approval for treating patients whose tumors exhibit microsatellite instability. With the advent of personalized cancer medicine, the portfolio of anti-tumor agents and companion diagnostic assays has experienced an unprecedented expansion, and a plethora of clinical trials are exploring the most effective treatments for the best-matching patients. In this context, data-driven approaches for optimizing clinical matching, reducing the cost of diagnostic testing, and improving the prediction of clinical phenotypes hold great promise for enhancing the clinical management of patients and maximize the value of precision oncology. However, all stakeholders must be aware of the important challenges that need to be overcome to reach these goals. This editorial aims at raising awareness about these challenges.

Artificial intelligence (AI), and more concretely its machine learning (ML) branch, can process large-scale and heterogeneous data sets to discern medically relevant patterns. ML is hence showing promise for making a positive impact in patient care. Pioneering works in the field of computer vision, and specifically in digital pathology, have demonstrated the enormous potential of ML models for improving the accuracy of diagnostic protocols using automated methods that require little human input. For instance, deep learning models can learn to predict molecular features of tumor samples, such as driver mutations or histological subtyping, based on features of histopathological images^1,2^. Approaches such as this could be scaled-up and incorporated in clinical pathways to streamline the time-consuming task of histological examination, and the generalization across tumor types and potential for accelerating clinical diagnoses through assisting expert pathologists have been supported by recent studies^3–5^. The field of diagnostic radiology is another area where the integration of ML techniques might have an impact on early cancer detection and diagnosis. Models trained on sufficiently large and well-annotated data sets of mammography or chest radiographs have demonstrated potential to predict the risk of developing breast^6^ or lung^7^ cancers by extracting information that is often imperceptible to the expert human eye, and to identify prognostic imaging biomarkers that can be associated with long-term patient outcome^8^. In non-invasive molecular testing, random forest algorithms applied on circulating microRNAs^9^ can accurately diagnose glioblastoma preoperatively, and other types of ML analyses applied to DNA methylation signatures measured in plasma cell-free nucleic acids achieve robust performance for tumor detection and classification^10^. To date, however, these models have been tested in retrospective studies, and their real-world impact for accelerating clinical workflows and increasing the rates of early cancer detection remains to be achieved prospectively.

On another clinical front, AI-powered decision support systems are increasingly getting better at optimizing treatment decision making for cancer patients. In addition to their role in drug discovery efforts, ML models integrating data on tumor growth kinetics, molecular profiling, and pharmacological properties could provide accurate prospective recommendations on the most effective therapeutic approaches for individual cancer patients—be it through drug repurposing, by identifying synergistic drug combinations, or in optimizing dosing schedules to maximize therapeutic efficacy. For this to be achieved, population-scale data sets containing well-annotated clinical and molecular information need to be made available^11,12^. Issues such as tumor heterogeneity and sampling bias, tumor evolution dynamics, or incomplete information on electronic medical records, among many other factors, represent major roadblocks for using and integrating these sources of data to build ML models, and are only partially solved by approaches increasing training set size (number of patients) at the expense of relevance (e.g., including patients with other cancer subtypes or treated with related drugs) or robustness of the data (e.g., merging clinical trials with different dosage schedules or even exploiting less-controlled data such as electronic health records). Even in the uncommon situations where large, carefully curated, and relevant data sets with low levels of noise are available, several fundamental methodological challenges currently limit ML approaches, including reliable inference of testable causality^13^, identifying the most suitable algorithm and features to solve a given problem^14^, learning from high-dimensional data sets^15^, or accurately estimating model generalization^16^. A related medical challenge is found when predicting patient response or resistance to anti-cancer treatments, not only for cytotoxic chemotherapies that form the backbone of most treatment regimens, but also for targeted therapies that either elicit unacceptable toxicities or achieve modest benefit even in the presence of the associated genomic marker. Here too, ML models are particularly promising for improving these predictions by identifying an optimal combination of patient features, which may include a range of non-genetic tumor features, if provided with appropriate input training data^17^. To overcome the limitation imposed by the paucity of large, well-annotated patient data sets, some studies have attempted to predict clinical phenotypes using ML models trained on response data from preclinical experimental systems of patient-derived xenografts^18^ or large-scale in vitro drug response studies^19^. However, despite the high value of preclinical models for drug development^20,21^, their general usefulness for precision oncology remains to be proven^22^. The problem of predicting optimal treatment approaches and response patterns remains unsolved, despite substantial efforts, as these models have not so far influenced clinical care in a significant way. Notwithstanding, this nascent field may hold great potential for advancing precision oncology.

The success of AI systems across medical domains will also depend on establishing a roadmap that delineates the step-wise integration of digital tools in the clinic^23^. There have been several initiatives proposing “best practice” guidelines to ensure that AI methods are developed and deployed in a way that maximizes the benefit for patients. On the model development front, these include recommendations for providing sufficient methodological detail on the development of algorithms, and encouraging sharing of data sets and code to enhance the transparency in the reporting of AI algorithms in medicine^11,12,24,25^ and allow other researchers to determine the rigor, quality, reproducibility, and generalizability of the findings^26–28^. Studies also need to adopt standardized guidelines for describing and reporting aspects related to the purpose and context of the “clinical need” that is being addressed, the quality of data used to train the models—including issues related to power calculation, labeling, model biases, etc.—measures of performance, outputs and framework for integration in clinical pathways and workflows, among others. Many of these issues have been approached by recent checklists intending to promote a standard and transparent reporting of clinical interventions involving ML approaches^29–31^. In addition, successful deployment of AI systems in the clinic will largely depend on the trust that end-users—clinicians and patients alike—have in the recommendations provided. To achieve this, it will be necessary to educate users on the working principles and degree of interpretability of AI systems, and to carefully define and test the interfaces that enable human–computer collaboration.

The application of AI to precision oncology is still in its infancy. In recent years we have witnessed a proliferation of proof-of-concept studies that offer a glimpse of what the next generation of precision oncology could look like. We have outlined here several challenges that need to be overcome for AI to make strides in medicine. We have also argued that the prospective application of these AI systems needs to follow a responsible path in collaboration with all stakeholders. Expectations are justifiably high given these initial studies, but true progress can only come from a deeper understanding of the discussed limitations. We look forward to seeing how AI may enhance precision oncology approaches and improve patient care around the world in the years to come.
